# Long-term distress in older patients with cancer: a longitudinal cohort study

**DOI:** 10.3399/bjgpopen19X101658

**Published:** 2019-09-04

**Authors:** Stephanie Dauphin, Leontien Jansen, Tine De Burghgraeve, Laura Deckx, Frank Buntinx, Marjan van den Akker

**Affiliations:** 1 PhD Student, Academic Center for General Practice, Department of Public Health and Primary Care, KU Leuven, Leuven, Belgium; 2 PhD Student, Academic Center for General Practice, Department of Public Health and Primary Care, KU Leuven, Leuven, Belgium; 3 Postdoctoral Research Fellow, Academic Center for General Practice, Department of Public Health and Primary Care, KU Leuven, Leuven, Belgium; 4 Postdoctoral Research Fellow, Primary Care Clinical Unit, Faculty of Medicine, The University of Queensland, Brisbane, Australia; 5 Emeritus Professor, Department of Family Medicine, School CAPHRI, Maastricht University, Maastricht, The Netherlands; 6 Emeritus Professor, Academic Center for General Practice, Department of Public Health and Primary Care, KU Leuven, Leuven, Belgium; 7 Professor, Institute of General Practice, Johann Wolfgang Goethe University, Frankfurt, Germany; 8 Professor, Academic Center for General Practice, Department of Public Health and Primary Care, KU Leuven, Leuven, Belgium; 9 Professor, Department of Family Medicine, School CAPHRI, Maastricht University, Maastricht, The Netherlands

**Keywords:** neoplasms, older patients, risk factors, long-term distress, primary health care

## Abstract

**Background:**

Receiving a cancer diagnosis can be a major life event which causes distress even years after primary treatment.

**Aim:**

To examine the prevalence of distress in older patients with cancer (OPCs) up until 5 years post-diagnosis, and identify predictors present at time of diagnosis. Results are compared with reference groups of middle-aged patients with cancer (MPCs) and older patients without a cancer diagnosis (OPs).

**Design & setting:**

OPCs, MPCs, and OPs participated in a longitudinal cohort study in Belgium and the Netherlands by filling in questionnaires at designated time points from 2010–2019.

**Method:**

Data from 541 patients were analysed using multivariable logistic regression analyses.

**Results:**

At baseline, 40% of OPCs, 37% of MPCs, and 17% of OPs reported distress. After 5 years, 35% of OPCs, 23% of MPCs, and 25% of OPs reported distress. No significant predictors for long-term distress in OPCs and OPs were found. For MPCs, it was found that baseline distress (odds ratio [OR] 2.94; 95% confidence intervals [CI] = 1.40 to 6.19) and baseline fatigue (OR 4.71; 95% CI = 1.81 to 12.31) predicted long-term distress.

**Conclusion:**

Distress is an important problem for people with cancer, with peaks at different moments after diagnosis. Feelings of distress are present shortly after diagnosis but they decrease quickly for the majority of patients. In the long term, however, OPCs in particular appear to be most at risk for distress. This warrants extra attention from primary healthcare professionals, such as GPs who are often patients’ first medical contact point. More research into risk factors occurring later in an illness trajectory might shed more light on predictors for development of long-term distress.

## How this fits in

Previous research has shown that long-term distress can be a serious problem for people with cancer. This longitudinal cohort study shows that this is also the case specifically for older patients (aged ≥70 years). Baseline variables, such as fatigue, functional status, distress, and loss of a partner did not predict long-term distress in these older patients. The authors suggest that primary healthcare professionals should monitor these patients more closely, preferably at 1 year post-diagnosis, when distress prevalence seems to increase.

## Introduction

A cancer diagnosis, along with the subsequent treatment and period of recovery, can be a disruptive event in people's lives, causing psychological distress. Reported prevalence of distress in patients varies, but tends to be relatively high.^1–9^ Distress in people with cancer has been associated with a lower quality of life.^7^ Most studies have focused on distress in the short term, usually up to 1 year after diagnosis and treatment. Less is known about long-term distress in cancer survivors, but research on this topic has been an increasing point of focus in recent years. Findings indicate that distress can persist for a long period after diagnosis and treatment, negatively affecting the quality of life of cancer survivors.^10–18^

The fastest growing group of patients with cancer and cancer survivors are older people. This is owing to the ageing population — as cancer is more common in older people — and to advances in early detection and cancer treatment.^19^ OPCs and cancer survivors might be particularly at risk of distress because cancer, as well as ageing-related factors, can be risk factors for distress. A diagnosis of, and treatment for, cancer is very stressful and often results in distress, but age-related problems such as functional impairment, loss of loved ones, fatigue, and poor psychological health are also known risk factors for developing distress.^18–23^

It remains unclear whether older or younger people suffer more from distress. Literature shows that the effect of age on psychological distress in healthy and in unwell people is unclear.^24^ Therefore, the focus on distress in OPCs is important because it can shed light on both the effect of age and the impact of the disease itself.

In this article, the aim was to disentangle the effects of ageing and cancer on distress by comparing prevalences of distress in OPCs (aged ≥70 years) with MPCs (aged 50–69 years; effect of ageing) and with OPs (aged ≥70 years; effect of cancer). The aim was to investigate the prevalence of distress in the period from the time of cancer diagnosis to 5 years after cancer diagnosis. To the authors' knowledge, this has not been studied before. The second aim was to identify baseline variables that predict distress after a 5-year period.

## Method

Data for this article were derived as part of the ongoing Kanker bij LIMburgse en Vlaams‐Brabantse Ouderen Project (KLIMOP), a Flemish–Dutch cohort study.^25^ OPCs and MPCs were recruited through hospitals. OPs were recruited by GPs. Patients with cancer had breast, gastrointestinal, lung, or prostate cancer. Inclusion and exclusion criteria are listed in Box 1. Data were collected at baseline (for patients with cancer, up to 3 months after cancer diagnosis), 6 months, 1 year, 3 years, and 5 years post-diagnosis. Data collection took place from 2010–2019 through face-to-face and self-administered questionnaires.

Box 1 **Inclusion and exclusion criteria for KLIMOP patients**

**Table:** 

Inclusion and exclusion criteria for KLIMOP patients	OPCs	Control group: OPs	Control group: MPCs
**Inclusion criteria**			
Signed the informed consent form	✓	✓	✓
Aged ≥70 years	✓	✓	
Aged 50–69 years			✓
Life expectancy >6 months	✓	✓	✓
Has an understanding of Dutch	✓	✓	✓
Interview possible within 3 months of diagnosis of cancer	✓		✓
**Exclusion criteria**			
Has a formal diagnosis of dementia	✓	✓	✓
Has a previous diagnosis of invasive cancer	✓	✓	✓
Is too ill to participate	✓	✓	✓
Diagnosed with a cancer type other than those listed^a^	✓	✓	✓

^a^Patients had breast, gastrointestinal, lung, or prostate cancer.

KLIMOP = Kanker bij LIMburgse en Vlaams‐Brabantse Ouderen Project. MPCs = middle-aged patients with cancer. OPs = older patients without a diagnosis of cancer. OPCs = older patients with cancer.

### Main outcome

Distress was measured using the Distress Barometer (DB). The DB is a combination of the Distress Thermometer (DT) and the Colored Complaint Scale (CCS).^7^ The DT is a single-item measure of distress, in the form of a sketch of a thermometer on which participants can indicate the level of distress they experienced in the past week, on a scale from 0–10, with 0 = no distress and 10 = extreme distress, with an accompanying colour scheme (green/0 = no distress to red/10 = extreme distress). The CCS is a five-point scale on which participants are asked to indicate whether several problems have burdened them lately. The problems assessed include: pain, other physical ailments, feeling nervous and/or tense, concentration and/or memory complaints, anxiety, concerns about a partner or family, grief, anger, existential issues, and other problems. The CCS is also colour-coded from green (0 = no burden) to red (5 = extreme burden). Lower scores on this five-point scale correspond to 0–1 points (answers: not at all = 0, a little = 0, and quite = 1), whereas higher scores correspond to a score of 4 (answer: a lot), or 5 (answer: very much). The Dutch version of the DB has been validated positively against the Hospital Anxiety and Depression Scale in Belgium (HADS). The DB was found to be more accurate with regard to stress detection than the HADS. Additionally, the authors identified a general DB cut-off score resulting in a dichotomous outcome of ‘present’ or ‘no present’ distress. Distress is present when patients rate their levels of distress ≥4 on the DT, combined with a total CCS score of ≥4.

### Fatigue

The fatigue subscale of the European Organization for Research and Treatment for Cancer Quality of Life Questionnaire (EORTC QLQ-C30) was used.^26^ Scores ranged from 0–100 and higher scores indicate increased fatigue. To determine a cut-off score, the general reference values of all patients and/or all stages from the EORTC reference manual was used.^27^ The top 25% of these scores were treated as indicative of patients suffering from significant fatigue, resulting in a cut-off score of >55.57.

### Functional status

In line with previous KLIMOP publications, functional status was conceptualised as being dependent on or independent of others in performing daily activities. This was measured with the Lawton Instrumental Activities of Daily Living scale (IADL) and the Katz Index of Independence in Activities of Daily Living (KATZ).^28–30^ These instruments are mostly used in older populations and measure basic daily activities, such as bathing and dressing (KATZ), as well as activities that support an independent lifestyle such as grocery shopping and doing laundry (IADL). Being functionally dependent was defined as scoring dependency in at least one of the IADL or KATZ activities.

### Loss of a spouse or partner

Data were collected about marital status directly from the participants. Loss of a spouse or partner was defined as being married or living together at baseline and being unmarried, divorced, or widowed at 5 years post-diagnosis.

### Statistical analysis

All statistical analyses were conducted using SPSS (version 24). Descriptive statistics were used to describe baseline characteristics. Continuous variables were dichotomised in order to reflect daily clinical reasoning. Groups were compared at baseline using χ² tests. By means of logistic regression analysis, a multivariable model was built to assess the possible influence of baseline variables on the presence of distress after a 5-year period. Based on literature and expert advice, the following variables were selected: baseline distress, baseline fatigue, baseline functional status, cancer stage, and the loss of a partner or spouse during follow-up. First, these determinants were analysed univariately per patient group in order to identify significant associations (*P*<0.01) with distress after a 5-year period. Significant associations were found for all the selected variables except cancer stage. This variable was, therefore, excluded from the multivariable model. Sex was controlled for in the model. The adjusted ORs are presented with corresponding 95% CIs. The analysis is based on a subset in which only the participants who (fully or partially) filled in the questionnaire at baseline and (fully or partially) at 5-year follow-up were included. In this subset, there were missing values. These missing values were handled with multiple imputation (five imputed datasets) through the use of the fully conditional specification approach.^31,32^ Missing values were imputed for the following variables: distress at 5-year follow-up, baseline distress, baseline fatigue, and baseline functional status. Participant dropout was analysed by comparing baseline characteristics of the participants who dropped out after baseline and participants who passed away during follow-up with baseline characteristics of those who participated up to 5-year follow up.

## Results

The baseline dataset comprised 1495 participants. After 5 years, 541 patients were available for follow-up: 104 OPCs, 271 MPCs, and 166 OPs (Table 1). Overall, the majority of participants in each patient group were women, had left school aged 15–18 years, and were married or living with a partner. In the MPC group, the mean age was 60 years (SD 5.3), in OPCs 75 years (SD 4.6), and in OPs 77 years (SD 5.1). The majority of patients with cancer had stage I or II, and received surgery and chemotherapy with or without a combination of other therapies. Twenty-one per cent of OPCs, 14% of MPCs, and 42% of OPs used ≥5 types of medication, an indicator for the prevalence of comorbidity. Differences between OPCs and the control groups are illustrated in Table 1.

**Table 1.** Baseline characteristics of final study population (*n* = 541)

	OPCs (*n* = 104)	MPCs (*n* = 271)	OPs (*n* = 166)	Difference between MPCs & OPCs	Difference between OPs & OPCs
	*n* (%)	*n* (%)	*n* (%)	*P* value	*P* value
**Women**	80 (76.9)	209 (77.1)	104 (62.7)	0.967	0.014
**Mean age, years (SD)**	75 (4.6)	60 (5.3)	77 (5.1)	–	–
**Marital status**				<0.005^c^	0.957
Married or living together	65 (62.5)	212 (78.5)	101 (60.6)		
Unmarried or divorced or widowed	37 (35.6)	57 (21.1)	62 (37.3)		
Other	2 (1.9)	1 (0.4)	3 (1.8)		
**Age leaving school**				<0.005^c^	0.830
≤14 years	24 (24)	25 (9.4)	36 (22.1)		
15–18 years	44 (44)	124 (46.8)	69 (42.3)		
≥19 years	32 (32)	116 (43.8)	58 (35.6)		
**Type of tumour**				0.529	<0.001^c^
Breast	78 (75)	186 (68.6)	–		
Gastrointestinal	20 (19.2)	60 (22.1)	–		
Lung	–	2 (0.7)	–		
Prostate	6 (5.8)	23 (8.5)	–		
**Medication use (≥5)**	22 (21.2)	39 (14.4)	70 (42.2)	0.112	<0.001^c^
**Distress (yes)**	42 (40.4)	101 (37.3)	28 (16.9)	0.674	<0.001^c^
**Fatigue (yes)**	15 (14.4)	33 (12.2)	8 (4.8)	0.689	<0.010^c^
**Functional status (dependent)**	66 (63.5)	125 (46)	80 (48.2)	<0.005c	0.014
**Cancer stage**				0.024	–
I & II	79 (89.8)	187 (78.9)	–		
III & IV	9 (10.2)^a^	50 (21.1)^b^	–		
**Treatment type**				0.028	–
Surgery only	14 (14.9)	44 (17.2)	–		
Surgery and radiotherapy or hormone therapy or both	15 (16)	73 (28.5)	–		
Surgery and chemotherapy with or without any combination of radiotherapy or hormone therapy or immunotherapy	65 (69.1)	139 (54.3)	–		

CI = confidence intervals. MPCs = middle-aged patients with cancer. OR = odds ratio. OPs = older patients without a diagnosis of cancer. OPCs = older patients with cancer. SD = standard deviation

^a^Available data, *n* = 88; missing data, *n* = 16. ^b^Available data, *n* = 237; missing data, *n* = 34. ^c^Statistically significant (*P*<0.01)

### Prevalence of distress

**Figure 1.**
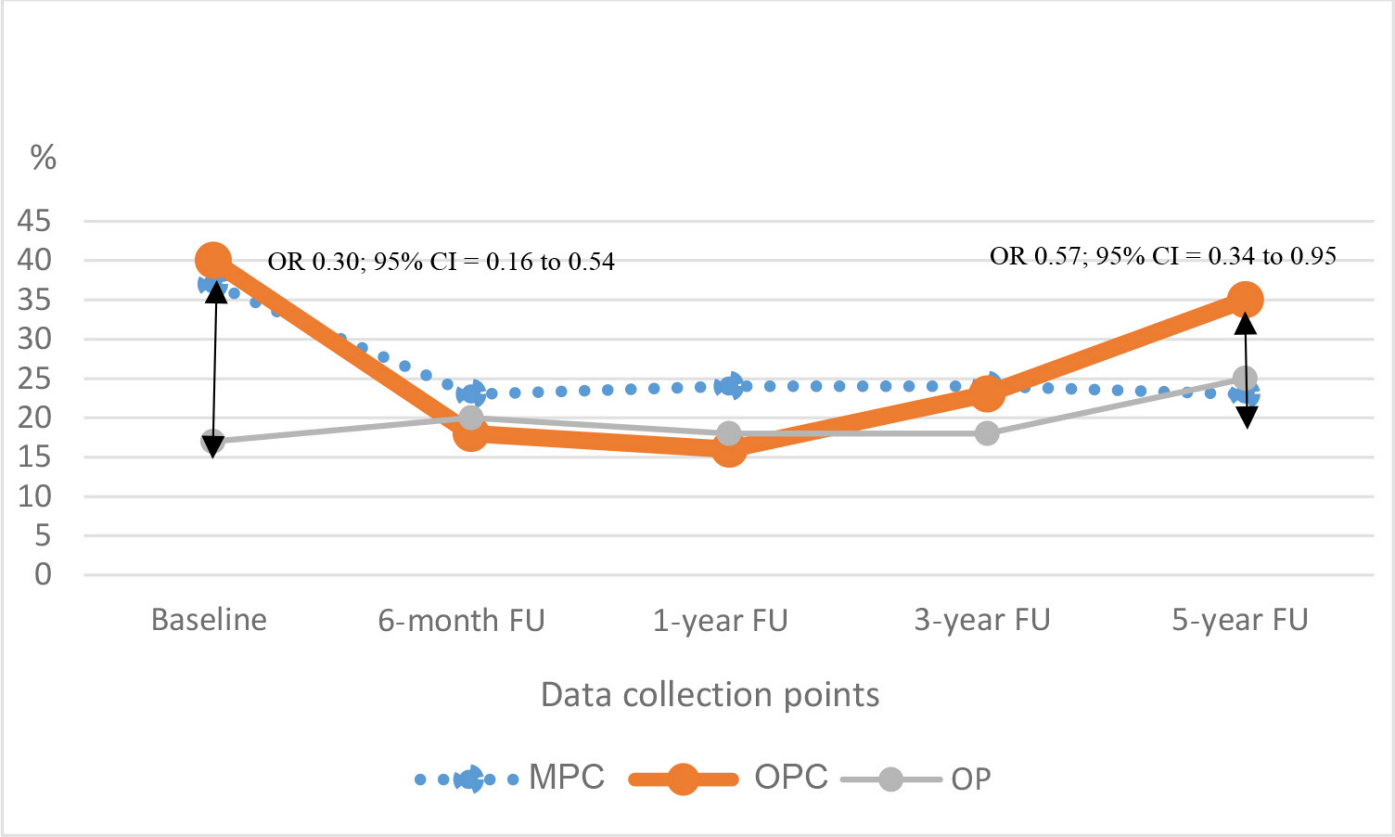
Prevalence of distress per patient group (*n* = 541) CI = confidence intervals. FU = follow-up. OR = odds ratio.

At baseline, 40% of OPCs, 37% of MPCs, and 17% of OPs reported distress. Within 6 months, the distress prevalence in patients with cancer decreased to a level roughly similar to baseline distress in OPs. One year after diagnosis, the prevalence of distress was lowest in the OPC group but after 5 years the proportion of OPCs reporting distress had increased to baseline level (35%). At 5-year follow-up, the prevalence of distress in MPCs and OPs was similar, at 23% and 25% respectively. When compared with OPCs, cross-sectional analysis revealed that, at baseline, OPs (OR 0.30; 95% CI = 0.16 to 0.54) and, after 5 years, MPCs (OR 0.57; 95% CI = 0.34 to 0.95) were significantly less likely to experience distress (Figure 1).

### Predictors of long-term distress

None of the included variables in the multivariable model were predictive of the presence of distress after 5 years in OPCs and OPs. For MPCs, it was found that baseline distress (OR 2.94; 95% CI = 1.40 to 6.19) and baseline fatigue (OR 4.71; 95% CI = 1.81 to 12.31) were predictors for distress after 5 years, while controlling for sex (Table 2).

**Table 2.** Multivariable logistic regression model with predictors for distress after 5 years, per patient group

	OPCs (*n* = 104)	MPCs (*n* = 271)	OPs (*n* = 166)
**Baseline predictors for distress after 5 years**	**aOR (95% CI)**	**aOR (CI 95% CI)**	**aOR (95% CI)**
Sex (reference category men)	2.19 (0.70 to 6.88)	1.16 (0.51 to 2.63)	0.88 (0.38 to 2.03)
Distress (reference category: no)	1.98 (0.68 to 5.80)	2.94 (1.40 to 6.19)^a^	1.84 (0.65 to 5.18)
Fatigue (reference category: no)	1.11 (0.25 to 5.00)	4.71 (1.81 to 12.31)^a^	2.20 (0.38 to 12.74)
Functional status (reference category: independent)	2.67 (0.95 to 7.49)	1.23 (0.95 to 7.49)	2.19 (0.94 to 5.06)
Loss of spouse/partner (reference category: no)	0.57 (0.12 to 2.71)	2.09 (0.62 to 6.96)	2.65 (0.90 to 7.81)

aOR = adjusted odds ratio. CI = confidence intervals. MPCs = middle-aged patients with cancer. OPs = older patients without a diagnosis of cancer. OPCs = older patients with cancer.

^a^Statistically significant.

### Extra analyses

At the time of 5-year follow-up, 263 patients (106 OPCs, 91 MPCs, and 66 OPs) had passed away and 616 patients (160 OPCs, 261 MPCs, and 195 OPs) were lost to follow-up (LTFU). The overall deceased rate of OPCs was almost two times higher than the control groups (27% OPCs, 14% MPCs, and 15% OPs). The overall dropout rates per patient group were quite similar (41% OPCs, 39% MPCs, and 43% OPs). Within each of the three patient groups, the proportion of patients with distress at baseline between the three LTFU categories (OPC, MPC, and OP) did not differ. When comparing baseline characteristics, death and loss to follow-up were mostly predicted by type of tumor (fewer in breast cancer), cancer stage (more if higher stage at diagnosis), and fatigue (more if fatigue was higher), as shown in Table 3. A complete case analysis was conducted and those results were compared with the results of the imputed missing dataset. In the complete case analysis, the significant difference between OPCs and MPCs after 5 years disappeared. In MPCs, the OR of fatigue almost doubled compared with the OR calculated in the imputed missing dataset. Additionally, losing a partner or spouse during follow-up turned into a significant predictor (OR 5.93, 95% CI = 1.46 to 24.14) for long-term distress in MCPs (Table 4).

**Table 3.** Baseline characteristics of participants lost to follow-up after 5 years and participants deceased after 5 years, compared with participants available for analyses after 5 years, per patient group (without imputed missing values)

	1: MPCs LTFU after 5 years (*n* = 261)	2: MPCs deceased after 5 years (*n* = 91)	3: MPCs available for analysis after 5 years (*n* = 271)	*P* value 1 versus 3	*P* value 2 versus 3	4: OPCs LTFU after 5 years (*n* = 160)	5: OPCs deceased after 5 years (*n* = 106)	6: OPCs available for analysis after 5 years (*n* = 104)	*P* value 4 versus 6	*P* value 5 versus 6	7: OPs LTFU after 5 years (*n* = 195)	8: OPs deceased after 5 years (*n* = 66)	9: OPs available to analysis after 5 years (*n* = 166)	*P* value 7 versus 9	*P* value 8 versus 9
	*n* (%)	*n* (%)	*n* (%)			*n* (%)	*n* (%)	*n* (%)			*n* (%)	*n* (%)	*n* (%)		
**Sex (women)**	182 (69.7)	38 (41.8)	209 (77.1)	0.05	<0.01^a^	111 (69.4)	51 (48.1)	80 (76.9)	0.180	<0.01^a^	121 (62.1)	35 (53)	104 (62.7)	0.907	0.177
**Mean age, years (SD)**	60 (5.5)	62 (5.5)	60 (5.3)	–	–	77 (4.8)	76 (5.4)	74 (4.6)	–	–	78 (5.4)	82 (5.8)	77 (5.1)	–	–
**Marital status**				0.329	0.183				0.045	0.576				0.232	0.389
Married or living together	202 (78)	69 (75.8)	212 (78.5)			78 (49.4)	67 (63.8)	65 (62.5)			127 (65.8)	36 (54.5)	101 (60.6)		
Unmarried or divorced or widowed	55 (21.2)	21 (23.1)	57 (21.1)			78 (49.4)	38 (36.2)	37 (35.6)			66 (34.2)	30 (45.5)	62 (37.3)		
Other	2 (0.8)	1 (1.1)	1 (0.4)			2 (1.3)	–	2 (1.9)			–	–	3 (1.8)		
**Age leaving school**				0.186	0.208				<0.01^a^	0.229				<0.01^a^	<0.01^a^
≤14 years	32 (12.7)	13 (14.6)	25 (9.4)			53 (34.2)	28 (27.2)	24 (24)			63 (33.3)	29 (43.9)	55 (23)		
15–18 years	128 (50.8)	45 (50.6)	124 (46.8)			79 (51)	53 (51.5)	44 (44)			91 (48.1)	29 (43.9)	108 (44)		
≥19 years	92 (36.5)	31 (34.8)	116 (43.8)			23 (14.8)	22 (21.4)	32 (32)			35 (18.5)	8 (12.1)	81 (33)		
**Type of tumour**				<0.01^a^	<0.01^a^				<0.01^a^	<0.01^a^					
Breast	146 (55.9)	13 (14.3)	186 (68.6)			85 (53.1)	34 (32.1)	78 (75)			–	–	–		
Gastrointestinal	78 (29.9)	31 (34.1)	60 (22.1)			56 (35)	38 (35.8)	20 (19.2)			–	–	–		
Lung	18 (6.9)	42 (46.2)	2 (0.7)			11 (6.9)	27 (25.5)	–			–	–	–		
Prostate	19 (7.3)	5 (5.5)	23 (8.5)			8 (5)	7 (6.6)	6 (5.8)			–	–	–		
**Distress (yes)**	84 (35.3)	33 (37.9)	95 (37.1)	0.675	0.891	40 (32.8)	25 (28.1)	*31* (*39.7*)	0.316	0.111	46 (24.7)	15 (23.1)	26 (16)	0.046	0.213
**Fatigue (yes)**	49 (19.1)	23 (25.3)	33 (12.2)	0.030	<0.01^a^	20 (15.2)	27 (27.8)	*11* (*13.9*)	0.807	0.026	18 (9.2)	9 (13.6)	8 (4.8)	0.106	0.020
**Functional status (dependent)**	122 (48.8)	50 (55.6)	121 (45.8)	0.500	0.111	93 (58.9)	68 (66)	66 (63.5)	0.456	0.700	105 (54.1)	45 (69.2)	79 (47.9)	0.238	<0.01^a^
**Cancer stage**				0.204	<0.01^a^				<0.01^a^	<0.01^a^				–	–
I & II	167 (73.9)	13 (24.5)	187 (78.9)			92 (74.2)	29 (42)	79 (89.8)			–	–	–		
III & IV	59 (26.1)	40 (75.5)	50 (21.1)			32 (25.8)	40 (58)	9 (10.2)			–	–	–		
**Treatment type**				0.440	<0.01^a^				0.406	<0.01^a^				–	–
Surgery only	43 (19.5)	5 (14.7)	44 (17.2)			17 (14.2)	3 (5.4)	14 (14.9)			–	–	–	–	
Surgery and RT or HT or both	71 (32.1)	22 (64.7)	73 (28.5)			28 523.3)	24 (42.9)	15 (16)			–	–	–	–	
Surgery and CT with or without any combination of RT or HT or IT	107 (48.4)	7 (20.6)	139 (54.3)			75 (62.5)	29 (51.8)	65 (69.1)			–	–	–	–	

CI = confidence interval. CT = chemotherapy. HT = hormone therapy. IT = immunotherapy. OR = odds ratio. LTFU = lost to follow-up. MPCs = middle-aged patients with cancer. OPCs = older patients with cancer. OPs = older patients without cancer. RT = radiotherapy. SD = standard deviation.

^a^Statistically significant (*P*<0.01).

**Table 4.** Complete case analysis: multivariable logistic regression model with predictors for distress after 5 years, per patient group

	OPCs(*n* = 48)	MPCs(*n* = 160)	OPs(*n* = 100)
**Baseline predictors for distress after 5 years**	**aOR (95% CI)**	**aOR (95% CI)**	**aOR (95% CI)**
Sex (reference category men)	2.34 (0.55 to 9.98)	1.43 (0.49 to 4.19)	0.57 (0.19 to 1.65)
Distress (reference category no)	2.92 (0.72 to 11.84)	**4.13 (1.68 to 10.17)**	2.21 (0.60 to 8.15)
Fatigue (reference category no)	1.00 (0.14 to 7.32)	**7.05 (2.31 to 21.56)**	2.47 (0.14 to 44.45)
Functional status (reference category independent)	1.77 (0.46 to 6.87)	0.85 (0.35 to 2.08)	1.62 (0.58 to 4.51)
Loss of spouse or partner (reference category no)	0.23 (0.02 to 2.31)	**5.93 (1.46 to 24.14)**	3.42 (0.87 to 13.42)

aOR = adjusted odds ratio. CI = confidence interval. MPCs = middle-aged patients with cancer. OPCs = older patients with cancer. OPs = older patients without cancer.

## Discussion

### Summary

The high prevalence of baseline distress in OPCs (40%) decreases in the first months after diagnosis but rises again after 1 year and continues to increase to levels similar to the baseline percentage (35%). OPs continue to have a low prevalence of distress over time (between 17% and 25%) whereas the distress prevalence in MPCs also decreases after diagnosis and then remains stable (from 37% to 23%). After 5 years, OPCs have the highest prevalence of distress. None of the assessed variables for distress in OPCs (such as distress, fatigue, functional status, and loss of loved ones) proved to be predictive of distress after 5 years in the study. The same is true of OPs. For MPCs, distress and fatigue at baseline were predictive of long-term distress.

### Strengths and limitations

This is a large-scale study with a longitudinal design that extends up to and including 5 years following diagnosis or inclusion. This allowed the prevalence of distress over a longer period to be explored. Another strength is the inclusion of the two control groups, facilitating an assessment of the influence of both age, and cancer diagnosis and treatment. The use of validated scales for measuring the psychosocial concepts also added to the robustness of this study.

An important limitation is the relatively high number of patients who dropped out and/or passed away during the study. This limited the analyses to a subset of the 541 patients still present at 5-year follow-up. This led to survivor bias: patients still participating after 5 years were more likely to have a favourable prognosis, were more highly educated, and were less fatigued in comparison with patients who dropped out and those who passed away. The results from the multiple imputation dataset differed slightly from the results of the complete case analysis. The difference could be attributed to the low number of MPCs in the complete case analysis who lost their partner or spouse or who had baseline fatigue. This has to be taken into account when interpreting the results of this patient group. The study only included breast, gastrointestinal, lung, and prostate cancer; as such, results cannot be generalised to patients with other types of cancer. The diversity of the sample can also be interpreted as a limitation because it does not allow for specific cancer-type results. Moreover, men are underrepresented in this study owing to the fact that the majority of patients with cancer in the sample are women with breast cancer, especially below the age of 70 (Table 1).

### Comparison with existing literature

Forty per cent of patients with cancer reported distress around the time of diagnosis. This is not a high number taking into account that estimates of clinically relevant distress in patients with cancer in other studies range between 35% and 62%.^2–5,7^ These prevalence estimates, however, are not specific to older or middle-aged adults. The prevalence of distress in older adults identified by Hurria *et al* ^20^ was similar (41%) to the present study’s baseline findings. The fact that their caseload with respect to tumour types was different and that they had a higher number of patients with metastases further corroborates the present study’s results: it indicates that this prevalence level is not underestimated owing to healthy user bias. At baseline, the percentage of distressed OPs is significantly lower than the percentage of distressed OPCs. This supports the supposition that the distress effect of the diagnosis is strong at that specific time. Several studies also attribute high levels of distress to the time of diagnosis.^33–35^ Percentages on long-term distress prevalence range between 20% and 33%.^12,15,36,37^ These numbers are somewhat similar to the present results. Dunn *et al* ^38^ found a decrease in distress over the course of 5 years in patients with colorectal cancer. Such a decrease is visible in the present study’s MPC group but not in the OPC group. In another study in older cancer survivors, one-third continued to suffer from cancer-related worries and fears.^15^ It cannot be confirmed whether the distress that 35% of the present study’s OPCs report is owing to their diagnosis, but the fact that it is higher than in OPs points to possible sequelae of cancer. A more recent study from the same authors in the same population, however, showed that long-term cancer worries can also be linked to general health concerns that are owing to ageing.^16^ The low distress prevalence in the OPs does not support that claim. The finding that, over time, the proportion of MPCs that experience distress is similar to or even lower than in OPCs is not always supported by previous studies. An increase in distress in OPCs is seen in the long term, whereas the distress prevalence level remains stable for MPCs. Previous studies usually show that younger patients with cancer are at higher risk for developing distress in comparison with older patients.^4,38–41^. However, a recent study in OPCs identified increasing age as a risk factor for depression and distress.^42^ Adding to the confusion in interpreting and comparing results are the different cut-offs for age groups in these specific articles and more widely throughout the literature. It is quite possible that the cut-off for older patients as aged ≥70 years (based on recommendations of the International Society of Geriatric Oncology)^43^ resulted in relatively lower proportions of MPCs reporting distress.

None of the assessed variables were predictive of long-term distress in OPCs and OPs, even though these selected predictors were consistently found to be long-term distress predictors in earlier studies.^18,20,21^ Baseline distress and baseline fatigue, however, were positively associated with long-term distress only in MPCs. A possible explanation for the absence of significant risk factors in OPCs could be that their long-term distress was not cancer-related, while baseline levels of distress were mostly related to the moment of diagnosis. Factors occurring later in the illness trajectory may have more influence on the development of long-term distress.

### Implications for research and practice

Healthcare professionals should be aware that OPCs may suffer from significant distress, which may persist over time. Primary healthcare professionals, such as GPs, should monitor this population more closely in order to detect or prevent possible feelings of distress. The results show that an appropriate time for this could be 1 year after diagnosis. This is usually the period at which patients finish primary treatment and transfer from hospital care to primary care. The absence of identifiable predictors for long-term distress in OPCs at time of diagnosis calls for more research. It is quite possible, however, that the development of long-term distress in this subpopulation is linked to factors occurring later in the illness trajectory.
